# Usefulness and engagement with a guided workbook intervention (WorkPlan) to support work related goals among cancer survivors

**DOI:** 10.1186/s40359-017-0203-2

**Published:** 2017-10-04

**Authors:** Lauren Schumacher, Maria Armaou, Pauline Rolf, Steven Sadhra, Andrew John Sutton, Anjali Zarkar, Elizabeth A. Grunfeld

**Affiliations:** 1 Coventry University, Centre for Innovative Research Across the Life Course, Coventry, UK; 2 University of Birmingham, Occupational and Environmental Medicine, Institute of Clinical Sciences, Birmingham, UK; 3 University of Leeds, Leeds Institute of Health Sciences, Leeds, UK; 4 University Hospitals Birmingham National Health Service Foundation Trust, Oncology, Queen Elizabeth Hospital, Birmingham, UK; 5 Birkbeck, University of London, Department of Psychological Sciences, London, UK

**Keywords:** Cancer, Oncology, Return to work, Intervention

## Abstract

**Background:**

Returning to work after cancer is associated with improved physical and psychological functioning, but managing this return can be a challenging process. A workbook based intervention (WorkPlan) was developed to support return-to-work among cancer survivors. The aim of this study was to explore how participants using the workbook engaged with the intervention and utilised the content of the intervention in their plan to return-to-work.

**Methods:**

As part of a feasibility randomised controlled trial, 23 participants from the intervention group were interviewed 4-weeks post intervention. Interviews focussed on intervention delivery and data was analysed using Framework analysis.

**Results:**

Participants revealed a sense of empowerment and changes in their outlook as they transitioned from patient to employee, citing the act of writing as a medium for creating their own return-to-work narrative. Participants found the generation of a return-to-work plan useful for identifying potential problems and solutions, which also served as a tool for aiding discussion with the employer on return-to-work. Additionally, participants reported feeling less uncertain and anxious about returning to work. Timing of the intervention in coordination with ongoing cancer treatments was crucial to perceived effectiveness; participants identified the sole or final treatment as the ideal time to receive the intervention.

**Conclusions:**

The self-guided workbook supports people diagnosed with cancer to build their communication and planning skills to successfully manage their return-to-work. Further research could examine how writing plays a role in this process.

**Trial registration:**

Current Controlled Trials ISRCTN56342476. Retrospectively registered 14 October 2015.

## Background

In the UK each year, over 120,000 people of working age are diagnosed with cancer [1]. Returning to work following illness is associated with improved physical and psychological functioning; conversely, being out of work is associated with reduced self-esteem and self-efficacy, a diminished belief in one’s ability to return-to-work, and has been shown to have a negative effect on physical and mental health [2–4]. Return-to-work rates differ across cancer types [5] and can take longer for survivors who received chemotherapy [6], those experiencing fatigue [7], or those whose workplaces are not supportive [8]. Returning to work can be a challenging process, especially when survivors report feeling anxious about continuing treatments and their perceived ability to perform work tasks [9].

Exploring the factors associated with delays in returning to work can be a complicated and individual process, as cancer type and treatment impact on physical symptoms, which impact on physical functioning, which in turn influences how an individual perceives their own health, which ultimately affects work outcomes [10]. However, a study that analysed patients by four specific cancer types demonstrated that after workplace accommodations and symptom management are taken into account, time to return-to-work could be predicted by patients’ beliefs about their cancer (such as the consequences of cancer) and their treatment (such as how the effects of cancer could be controlled or accommodated for in the workplace) [5]. As such, there is a need for more research to support cancer survivors to return-to-work, with a specific need for return-to-work interventions that address social, clinical, and work-related aspects of the process [11].

Furthermore, there is a need for more research aimed at understanding cancer survivors’ engagement with interventions to support them in returning to work and undertaking a qualitative analysis of intervention engagement is crucial to shedding light onto why an intervention succeeds and how to further improve the content, or conversely, to understand why an intervention does not perform as expected [12]. Qualitative analysis of engagement can provide rich data on how participants experience and apply an intervention, revealing data beyond just *effectiveness*, and can identify what participants want and need from interventions and how they can be developed and best delivered [13, 14].

Existing literature evaluating print-based interventions have examined how participants engaged with the material through reading and acquiring knowledge, discussing the material with others, writing personal diaries, and retaining the written materials for future reference [15, 16]. But there is little research examining how participants engage with an intervention in terms of the application or implementation of the material to their individual situations. This study aimed to explore how participants using *WorkPlan* (a workbook based intervention aimed at improving work-related outcomes in cancer survivors) engaged with the intervention and utilised the content of the intervention in their plan to return-to-work.

## Methods

Ethical approval for this study has been obtained from West Midlands–Solihull (National Research Ethics Service) Research Ethics Committee (Reference: 15/WM/0166). This research was informed by the ‘The Consolidated Criteria for Reporting Qualitative Studies (COREQ) 32 item checklist’ [17]. Participants were recruited to the study from five UK hospital sites as part of a larger study examining the feasibility of a randomised controlled trial of a workbook based intervention (WorkPlan) to support return-to-work among cancer survivors. The intervention package and the feasibility trial have been reported [18]; in summary, the workbook intervention was theoretically led and grounded in the self-regulation model and goal setting theory. The workbook contained four chapters to be completed weekly and included activities to encourage thoughts/beliefs about cancer and how it could affect work, develop goals around return-to-work with small achievable steps, culminating in the creation of a return-to-work plan: a personalised document incorporating actions to be taken to aid the transition back to work. Participants also received two telephone support calls at weeks two and four of the intervention period. These phone calls gave participants the opportunity to discuss their progress, ask questions about items they found difficult, and seek clarification on any of the workbook content; the research team also highlighted the return-to-work plan in the workbook. All participants included in this interview study had been randomised to the intervention arm of the trial. At the time of recruitment, participants met inclusion criteria of at least two weeks posttreatment initiation and were identified through breast, gynaecological, colorectal, or urological cancer clinics and through multidisciplinary team meetings. Recruitment and study materials were translated into the five most commonly spoken languages among people of working age within the recruitment area and interpreters were available during the interviews if required. However, despite recruitment materials in multiple languages, no patients were recruited into the study who required either translated materials or an interpreter and all interviews were conducted in English. Potential participants were provided with information about the study and given contact details, and asked to contact the research team by telephone or email if they were interested in participating. All participants completed a baseline assessment in person or over the telephone where they discussed the nature of their work and the researchers introduced the intervention. Following completion of the four week workbook intervention, eligible participants were contacted by post and telephone and invited to be interviewed at one of the hospital sites or over the telephone. Participants were approached sequentially until the recruitment target of 20 was reached; 26 participants were approached, 1 declined to participate (no reason given) and 2 were unable to take part due to scheduling conflicts.

The interview schedule was developed through a review of previous research and discussion with a team of health psychologists and oncological clinical nurse specialists. The interview schedule focussed on how the intervention was delivered, aspects of the intervention individuals found useful, and general perceptions of cancer, work values and perceptions of the potential impact of their cancer on work. Pilot testing of the interview schedule was undertaken with cancer survivors who were not participants in the current study and had reviewed the workbook. The schedule was not rigidly adhered to, allowing discussion of issues that were important to the participants, who were encouraged to talk openly about issues of relevance to them. Interviews lasted on average 70 min (range 33 to 132 min). All interviews were conducted privately over the telephone and audio recorded (with permission) by two MSc qualified female research assistants (LS and PW) and a female PhD student (HM); all interviewers had previous experience conducting qualitative interviews. At time of interview all participants were familiar with the interviewers as they had been recruited by them and completed baseline assessment measures with them.

### Analysis plan

Interviews were recorded and transcribed verbatim. To maintain anonymity each participant was assigned a pseudonym. Accuracy of the transcripts was checked against the original recordings. A “framework” analysis approach [19] was used. Framework is a flexible approach utilising an iterative process and the approach primarily follows the constant comparison method [20]. Framework analysis was chosen as it is a systematic approach to qualitative analysis that guides researchers through a process of familiarisation with the data, the creation and application of a framework, the charting of data by both theme and individual participant, and the interpretation of the entire dataset [21]. Following the completion of all the interviews the transcripts were analysed by LS and MA by noting relevant units of meaning and creating free codes. Following on from this the free codes were then grouped into coherent themes. Once themes were identified for each participant they were integrated across participants to generate a list of super-ordinate themes that captured the participants’ shared experiences. Six (26%) transcripts were independently analysed by MA; the researchers met to discuss the analysis. Only minor differences in researcher perspective emerged regarding how transcript passages were assigned to themes and these were resolved by mutual agreement. LS completed 75% of the data analysis, with frequent discussions with MA and EG to confirm the analysis was valid.

## Results

Twenty-three participants were interviewed, of whom 16 were female and 7 were male. Participants ranged in age from 25 to 65 with a mean age of 50; 12 participants had a diagnosis of breast cancer, 7 with urological cancer, 3 with bowel cancer, and 1 with gynaecological cancer. Twenty participants described themselves as employed and 3 as self-employed, with 11 participants working in the public administration, education, and health industry, 7 in the distribution, hotels, and restaurants industry, 1 in finance, and 4 in other services. Twenty-two participants returned to work during the 12 months of data collection for the study; 9 participants returned within 12 weeks after intervention delivery, 6 within 13-24 weeks, 3 within 25-36 weeks, and 4 within 37-52 weeks (see Table 1).

**Table 1:** Participant demographics

Age, mean (range)	50 (25–65) years
Gender, n, %	
Female	16 (70%)
Male	7 (30%)
Ethnicity, n, %	
White	20 (87%)
Caribbean	1 (4%)
Indian	1 (4%)
Information Not Provided	1 (4%)
Marital Status, n, %	
Married or Living with Partner	14 (61%)
Divorced or Separated	5 (22%)
Single and Never Married	3 (13%)
Information not Provided	1 (4%)
Cancer Diagnosis, n, %	
Breast	12 (52%)
Urological	7 (30%)
Bowel	3 (13%)
Gynaecological	1 (4%)
Work Status, n, %	
Full-time	14 (61%)
Part-time	6 (26%)
Self employed	3 (13%)
Industry, n, %	
Public Administration, Education, and Health	11 (48%)
Distribution, Hotels, and Restaurants	7 (30%)
Finance	1 (4%)
Other Services	4 (17%)
Time from Intervention Delivery to Return-to-Work, n, %	
1–12 Weeks	9
13–24 Weeks	6
25–36 Weeks	3
37–52 Weeks	4
No Return-to-Work During Study	1

Interviews revealed rich accounts of how engaging with the workbook represented a transition towards a future at work and the steps that took place to make that a reality. Themes around this transition included *Impact on Patient Outlook*, whereby participants recognised changes within themselves that facilitated the start of the return-to-work process, which was enabled by the *Impact of Writing*. The workbook allowed participants to begin *Planning for the Future* by drafting specific return-to-work plans and was useful in *Supporting Communication with Employers*. Finally, *Timing of the intervention was the key to its perceived effectiveness*, with the majority of participants expressing a clear preference for when the intervention should be delivered.

### Impact on patient outlook

Twelve participants discussed the transition they experienced in their perceptions of themselves, their future, and how they could prepare to for an eventual return-to-work. Participants explained how cancer treatment was something done to them, not something they were able to play an active part in, describing a sense of victimhood as they progressed from diagnosis through treatment and coping with ensuing side effects. Working on the workbook gave participants the opportunity to regain control of their bodies and their lives, instilling confidence and enabling them to picture a future after cancer.

“The whole exercise overall probably gives you a sense of control, gives you control back, and everybody likes control, and one of the issues of having cancer and cancer treatment is that total lack of control, that you’re suddenly having things done to you, and your body is doing things that you can’t control. So that, particularly, some of the exercises are obviously a major effort in helping you gain control again.” (Participant 12).

“I thought it was a useful tool to get you thinking about it and taking ownership of stuff because you can feel a bit like a bit of a victim if you're not careful, and giving you an awareness that there's stuff that you can do to take back control because you do feel, when you're a patient, that actually stuff is being done to you.” (Participant 24).

Many participants reported how the workbook helped them organise their thoughts about return-to-work, bringing clarity to their needs and priorities and alleviating uncertainty. Beyond the concerns for managing fatigue and travel, the workbook prompted participants to address if they even wanted to return-to-work at all. Participants felt overwhelmed by just beginning to consider a return-to-work and were empowered by the workbook to set goals, identify barriers, and reflect on the small details that would make a return manageable and successful.

“I like the idea that there's something there to support a return-to-work, because, well specifically for me, I am currently at a very confused point in my life, where you need something I think, when you've been out of work for so long, to sort of, pin it all down to and put everything in the one place, which I think does really well, this helps me to do really well.” (Participant 16).

“I liked the fact that it broke it down into small bits and that, actually, it focused the mind… It focused my mind, anyway, on actually coming up with a plan and what I actually thought. I think sometimes you have big thoughts, but they’re that big, you don’t tend to break them down. So, I quite like the fact that it breaks it, step by step, into, ‘What do I actually think? What’s a barrier? What’s going to stop me from doing this?’” (Participant 36).

A small number of participants noted that working on the workbook was in itself a way of preparing for return-to-work. After being on sick leave for several months, participants perceived a stagnation in their cognitive functions and working on the workbook was just the sort of task to engage their minds by thinking about the process of returning to work, the work environment, and the mental demands of work.

“It’s like doing work, having something that’s not just nothing, just sitting around waiting, because after the length of time you’ve been on treatment that’s pretty much what a lot of it is… to actually have something constructive.” (Participant 40).

### Impact of writing

Participants described feeling unclear and uncertain about the process of returning to work. A total of fifteen participants spoke at length on the impact of writing on their overall engagement with the intervention. The act of writing was a tool to help focus all their thoughts about work and how to manage their return; by writing down these thoughts, participants were able to focus in on what was important by unravelling the ruminations.

“Focus, creating a focus. It is very easy to have everything in your head, it all swimming around in your head and going in circles. Whereas if you actually write it down you can often find at least a start point and then go from it.” (Participant 09).

“I found that actually writing it on the piece of paper was useful. Instead of having it airy fairy in my head having thought thoroughly about it and having a plan in my head then the exercise of putting it down on paper clarified my thoughts.” (Participant 15).

The act of writing also allowed participants to voice their thoughts and emotions and take ownership of them, thereby giving them validity and meaning. Instead of just reading the intervention content, participants crafted a narrative using their own words; in doing so, this new content became immediate and personal.

“I thought that was quite useful for actually jotting stuff down because I think once you physically write it, it's there on the page and you have to own it. In your own handwriting, it makes it more personal.” (Participant 24).

“Really useful, because it’s handy to be able to write your thoughts down. It’s not just a booklet that you read; it’s something that becomes yours by you putting your bit in.” (Participant 36).

The act of writing created a space where participants could imagine themselves back at work and brainstorm about the realities of being back. Simply reading the provided content was not sufficient for participants to apply it to themselves; participants were able to come up with ideas to solve return-to-work challenges by writing down different options and evaluating how effective they might be. Several participants found that writing in this way helped relieve the anxieties they were feeling about their return-to-work challenges.

“I find if I write things down it stops things going round and round. Being able to write it down, look at it and think, ‘Actually that makes sense.’ Then not just writing down the problem but how am I going to cope with it. Actually having to write something down that does help me.” (Participant 09).

“It was just asking me to explain what tends to happen when I'm really fatigued and how that makes me feel, which is nice, and actually I think sometimes, it's enough to have something like that and I think you just write down what you feel and how helpful you find it, that in itself, then reading that over again in itself, kind of helps you form a solution. Does that make sense? Just being able to write it down and sort of put pen to paper and really connect with it makes it easier to think of a solution, rather than think of a solution to this problem now, you know?” (Participant 16).

### Planning for the future

The final section of the booklet culminated in the My Plan Worksheet, the document which guided participants to incorporate all previous content into a personalised return-to-work plan. My Plan Worksheet invited participants to specify when they intend to return-to-work and on what hours and workdays, any concerns or support needs, as well as the precise work tasks they are and are not able to do.

The My Plan Worksheet was specifically mentioned by eleven participants as beneficial to providing definition and precision to how and when they would return to their job and working environment; this level of specificity was described to relieve their anxiety about returning to work.

“I think I found this [My Plan] probably the most useful bit, the closest to physically doing something about getting back to work and that sort of thing… I like the idea of pulling it all together to actually get back to doing something.” (Participant 09).

“I think when you get the My Plan Worksheet, it’s really good, because it breaks it down into, ‘What do I need to change? What am I going to need to think about?’ Shifts, travel, work environment, job. All of those things. ‘What am I going to be able to do? What am I not going to be able to do?’” (Participant 36).

Participants found the booklet was a useful guide to planning and preparing their return-to-work, often allowing participants to imagine the potential problems that could arise, thereby making plans to prevent or lessen their impact at work. Participants felt prepared by considering all daily work activities, work-related tasks, and potential events at work, such as coping with co-workers’ reactions, and how they might respond to them. In this manner they were able to engage in mental role play to rehearse how to respond, gaining strength and confidence in their abilities.

“I think that was quite a useful thing for me to think about, about possibilities. You can role play in your head what you might do should things come up. I think the perception is that you feel that you're just going to go back to work and it's all going to be as it was. I think being aware that there might be some barriers means that you're not quite so stymied when one hits you.” (Participant 24).

“It gives you that mental strength to face challenges and future emotions at work, it puts you there before that and when you do the work plan you’re trying to make promises to yourself. I think that’s the most useful thing.” (Participant 56).

### Supporting communication with employers

The workbook was described by twelve participants as a platform for initiating contact with employers and setting out how these discussions would take place. Five of these participants were closer to returning to work than their counterparts and were able to use the workbook content in actual meetings with their employer.

Participants revealed that they felt empowered to reach out to their employer to communicate with them about returning to work. Some participants stated that it had never occurred to them that this would be beneficial, that they would want to initiate this communication, or that there would be people, such as employers or colleagues, who would want to listen and speak with them.

“I hadn’t really had any discussions with my managers or anything about going back to work, and there won’t be, I mean, you have to generate that. They’re actually quite receptive on the whole, but they haven’t thought about it much either… So in a sense it’s up to you to come back and make suggestions, so that’s why I think it was very helpful, because it was a list of things I could take to my manager.” (Participant 12).

Participants found that the knowledge they gained from the workbook, especially the material around creating a return-to-work plan, was an ideal approach to communicating with their employers. The My Plan Worksheet was described as an excellent tool for guiding discussions with employers and for joint planning sessions for managing their return.

“It [My Plan Worksheet] was going to be useful to show to my manager. I was going to use it as a basis for a meeting with her. It was also useful just to jot down thoughts about this business about the duties I will do and won't do I thought were quite useful… Because it's on an official sheet, if I take it to work and show them then they can see that I'm taking it seriously.” (Participant 24).

“When I have a meeting with them [employer], I think the booklet might be helpful for reminders for me to think, ‘I want to discuss this with them, I want to discuss that with them’ and see what support could be put in place.” (Participant 29).

Five participants were able to use knowledge they had gained from the workbook in meetings with their employers, using the My Plan Worksheet as a guide. Positive outcomes were reported from these interactions, with participants feeling confident in how to conduct themselves in meetings with the certainty of how to portray their specific return-to-work needs.

“We were able to talk about what duties I could and couldn’t do without some help. I didn’t say I won’t do anything, I just said, ‘I will need some help doing stuff.’ Rather than, ‘I will not do.’… I was able to go into the meeting instead of umming and ahhing I know exactly what I wanted to do and exactly what I needed to say.” (Participant 02).

Participants were able to establish firm plans with their employers and solidify how their return would be managed, focusing on the details of phased returns, works tasks they would need support in accomplishing, and how their support plans would be monitored and assessed.

“We were able to do risk assessments on what they [employer] thought I couldn’t do and what I thought I couldn’t do… They are going to look at the risk assessments in two months and reassess them if I am a lot healthier…Then we have gone on a phased return. The first week between us we decided we would start with 10 hours and then work up to my full 30 hour contract over a period of a month. Every two weeks we are having an interview to make sure I am coping well with it.” (Participant 02).

### Timing of the intervention was the key to its perceived effectiveness.

Four participants reported that they received the workbook at the best time for them and were able to obtain the maximum benefit from it. Amongst these four participants, two had completed their sole treatment for urological cancer and two were patients with breast cancer in the midst of completing radiotherapy as the final treatment in their regimen.

“I think just before you start radiotherapy is quite a good time because you're starting to think, ‘Well this is the last section now and the last part of my treatment,’ or whatever. I think if I'd have had it before surgery or anything like that, then it would have felt too long away. I wouldn't have been able to get my head around that… because you're coming towards the end of your active treatment so then work to know what are your thoughts on coming back…” (Participant 13).

Five participants received the workbook while undergoing initial treatments for breast (4) and bowel (1) cancers, four of whom were undergoing chemotherapy. These participants revealed that their focus was devoted to getting through their treatments and they simply could not devote their cognitive resources to thinking about work, something which felt very far away.

“I’d probably give this book to people nearer the time of returning to work, and if it’s a four-week programme, maybe four, five, six weeks before they go back to work might be a bit more useful. Even a couple of months, I think, before they go back to work… So, once you’ve been through the bulk of your treatment and you really are a couple of months away, it might be more beneficial then.” (Participant 30).

Three men with urological cancers reported receiving the workbook too late into their treatment process to acquire as much, or any, benefit from it.

“I think the problem was that I didn’t have quite enough time, because I was on it [the booklet] less than a week before I went back to work, and probably should have started it a couple of weeks, to really get the best out of it.” (Participant 12).

Four participants received the workbook as they were nearing the end of chemotherapy (3) or were about to start their third and final treatment (1). These participants described initially struggling with the workbook, similarly to the participants who felt they received the workbook too early; however, as their treatments progressed and their physical health improved, they felt more able to engage with the material. Participants also reported that treatment progression naturally led to wanting to engage with the material, as they were beginning to have more thoughts about finishing treatment and returning to work.

“When I wasn't feeling great, the thought of having this plan to work through, it was hard work. It was hard work to do it and to actually apply my head to actually think about things, because you know, the chemo affected my head and everything, and also the thought of going back to work was so abstract … Now that I'm feeling better, I feel more able to tackle the thought of going back to work, and so for me, definitely now that I'm sort of on the road to recovery, it feels like a better time to have the book and to work through it, but it might not be the same everybody.” (Participant 16).

## Discussion

This study aimed to explore how participants using *WorkPlan* engaged with the intervention and utilised the content of the intervention in their plan to return-to-work. Participants revealed how the workbook guided them to return-to-work through pre-preparation, planning, communicating their return-to-work needs, and in some cases, establishing their return-to-work plans. During their interviews, participants noted changes in their outlook as they transitioned from cancer treatment to returning to work: regaining control of their lives, creating a focus to their thoughts and ideas, which helped prepare participants for the mental challenges of reengaging at work. Experiencing a loss of control is not unique to the participants of the current study and patients reporting lower levels of perceived control tend to experience increased psychological distress and decreased adaptation, including aspects of depression, anxiety, mood, and quality of life [22–24]. Other studies have demonstrated how patients use a repertoire of strategies to regain control, often beginning with small acts of responsibility, such as cleaning or work tasks completed over the phone [25], building into mental efforts (positive thinking, meditation, etc.), lifestyle changes, information seeking, and trying to control side effects from treatment [26]. Acts such as these are reported to be a method of empowerment in women living with breast cancer [27]. Participants in the current study described the workbook as a way of making them aware of their transition from being a patient to someone who is able to make an active difference in their life.

Participants from the current study readily revealed how the physical act of writing in the workbook aided them in organising their thoughts, enabling them to plan for the future. The notion of expressive writing, or the formation of a written narrative exploring the emotional aspects of a personal experience, has been well documented in its associations with decreases in psychological distress and healthcare utilisation and improvements in self-reported physical health, immune functioning, and general psychological well-being [28–30]. Additionally, expressive writing has benefited cancer populations by leading to improvements in pain severity and decreases in healthcare utilisation [31] and improvements in psychological functioning and quality of life [32]. It is proposed that expressive writing allows an individual to create a coherent narrative of challenging life events through the combination of their own thoughts and emotions; this construction acts as a mental summary of the event, allowing it to be stored and coped with more effectively [33, 34]. Although the workbook was not intended as an expressive writing task, it nonetheless allowed participants to explore thoughts and emotions that they might not yet have had the opportunity to investigate with healthcare professionals, friends, or family.

Participants described how the act of writing helped to stop the flow of intrusive circular thoughts and worries about returning to work. Expressive writing has been found to decrease the more negative, self-judgemental aspects of rumination, as well as symptoms of depression. The authors proposed that spending time thinking about problems in a non-judgemental way enabled individuals to explore a wide variety of potential solutions [35]. Although it cannot be inferred that the workbook in the current study acted as a protection against depression, it is possible that participants were able to stave off the more negative qualities of rumination. Participants in the current study found a print version of the workbook a particularly effective way to engage with both the material and their thoughts. Although a group of U.S. Afghanistan and Iraq war veterans found an online expressive writing intervention effective in decreasing physical complaints, anger, and distress [36], previous research with cancer patients indicated that a print intervention was deemed more acceptable than accessing materials online [37].

Participants in the current study reported how the workbook gave them the knowledge and skills to engage and communicate with their employers. When employers establish contact, it fosters a sense of goodwill and trust and reminds employees they have not been forgotten or ignored, often encouraging a sense of collaboration and leading to a successful return-to-work [38–41]. Despite this, some employers cited feeling uncertain regarding when and how to contact a sick-listed employee [42]. Maintaining communication with employers during sick leave has been shown to be integral to returning to work after a long term sickness absence; successful return-to-work interventions were found to more likely contain an element of communication between employers and employees [43] and employees’ return-to-work was quicker when supervisors maintained regular contact with the sick listed employee [44, 45].

Employees who develop a return-to-work plan have described them as useful [46], a way of regaining control [47], and a structured way of gradually increasing work tasks and therefore self-confidence [48]. This is not dissimilar to the participants in the current study, who found the act of planning essential for outlining their personal work priorities, areas of perceived difficulties and ways to overcome them, and how they wanted their work to fit into their post-cancer treatment life.

Participants in the current study noted that how the workbook coincided with their treatment either supported or limited its effectiveness. As participants in the current study were recruited across four different cancer types, treatment options varied. Whereas participants with urological cancers were typically treated with surgery only, sometimes in combination with radiotherapy [49, 50], participants with breast, bowel, or gynaecological cancers typically underwent a combination of at least two, if not all of the following: surgery, chemotherapy, and radiotherapy [51–56]. Although most participants found that the workbook complemented the end of their treatments, some patients reported receiving the workbook too late to obtain as much benefit as they would have liked and others, who were in the midst of chemotherapy, not only reported receiving the workbook too early but also described difficulties engaging with the content due to side effects from their treatment. Participants across studies have cited how their chemotherapy contributed to diminished cognitive skills, in areas such as memory, attention, decision making, verbal ability, information processing, and multitasking; these cognitive changes were often perceived as a barrier to returning to work [57, 58] and likely made working on the workbook challenging for participants of the current study.

The results of this study provide insight into how a self-guided workbook can facilitate cancer patients to plan and direct their return-to-work. There was a large percentage of women in the study and, as mentioned earlier, breast cancer treatment times can vary significantly to other cancer types, especially that of urological cancers. Another limitation was the brief reflection period of approximately 4 weeks post intervention delivery. Although 9 participants returned to work within 12 weeks of receiving the intervention, 13 participants did not return-to-work until 13-52 weeks later and were unable to comment on how they had applied the workbook to their individual situations. Participants also received two telephone support calls while they were completing the intervention; although the intention of the telephone calls was to support participants who may have had questions about the workbook, they could have inadvertently encouraged engagement with the intervention by reminding them to complete the activities. Potential bias could also exist due to the relationship that the researchers developed with participants, from the initial baseline assessment, the telephone calls, and the interview itself. Although the research team informed participants this was a feasibility study and as such it was vital to discover if improvements were needed to the workbook, participants may have felt inclined to give only positive feedback.

Future research could further explore the role of writing and how the physical act of putting words on the page aids planning a return-to-work, perhaps comparing it to a digital element. Additionally, participants should be followed up for a longer time period to explore how the workbook shaped and influenced their actual return-to-work, possibly exploring how the workbook can be utilised to support further workplace accommodations after the initial return period. Future interventions conducted with cancer patients should be mindful to the timing of the delivery to coincide with the start of the final or only treatment, perhaps avoiding the chemotherapy period altogether.

## Conclusions

This self-guided workbook supports people diagnosed with cancer to prepare for returning to work by creating a space to envisage and construct a future at work, building on communication and planning skills to brainstorm ways to achieve this goal.
